# Insights into the Anti-Aging Prevention and Diagnostic Medicine and Healthcare

**DOI:** 10.3390/diagnostics12040819

**Published:** 2022-03-26

**Authors:** Seung-Cheol Ok

**Affiliations:** Department of Special Physical Education, International University of Korea, Jinju 52833, Korea; 41thok@hanmail.net; Tel.: +82-55-751-8365

**Keywords:** anti-aging, anti-aging medicine, diagnostics, healthcare, aging

## Abstract

Aging is an irreversible and natural phenomenon that occurs as a person ages. Anti-aging medicine applies advanced science and medical technology to early detection, prevention, treatment, and reversal of age-related dysfunctions, disorders, and diseases. Therefore, anti-aging diagnostic medicine and healthcare are important factors in helping the elderly population lead healthy and active lives. However, it is challenging to diagnose various aging and related diseases accurately through various forms of anti-aging diagnostic medicine and health management. It may not be treated appropriately, so many older people are making various efforts to prevent aging themselves in advance. Therefore, anti-aging medicine and health care have been developed in various forms, from health checkups to alternative medicine and biophysical technology beyond simple clinical medicine, and are being applied to demand the needs of the elderly. This review intends to explore and characterize various applications related to anti-aging medicine and healthcare in the elderly. In addition, economic, medical, and ethical considerations arising from the relationship between the increase in the elderly population and the continuous development of anti-aging medicine can be considered.

## 1. Introduction

Aging is a natural phenomenon caused by the gradual deterioration of biological and physiological functions as a person ages [1,2]. At the biological level, aging is caused by accumulating various molecular and cellular damages over time. Accumulation of the development of cell degeneration may ultimately lead to death by diminished physical and mental capacity, induction of illness, and increased risk [3,4,5]. The cell degeneration effects can be explained by various factors and can be classified as follows: Internal factors are cell changes due to inflammation, hormone deficiency, and external factors are smoking, drinking, and inappropriate intake of nutrients. Therefore, aging reflects the biological reality of cells due to the effects of various factors on the body and has an inherent dynamism that cannot be controlled.

Anti-aging, which is generally used as the opposite concept of aging, can be defined as a kind of science used to prolong the lifespan and delay aging while maintaining the physical, functional, and aesthetic beauty of the body. However, beyond this general concept, the definition of anti-aging is rather complicated [6,7,8,9]. Anti-aging focuses on technical aspects that can delay, prevent, or reverse the aging process in the scientific community. In the medical community, anti-aging means early detection, prevention, and treatment of age-related diseases. It is different from the point of view of the scientific community that deals with the aging process itself, and various strategies and treatment methods can be used. Although it is interpreted as research and clinical approaches to anti-aging from a slightly different point of view, it has the same character as the ultimate aim of maintaining physical and mental health.

In particular, anti-aging diagnostic and medicine, which is part of medical and healthcare, applies advanced science and medical technology for the early detection, prevention, treatment, and reversal of age-related dysfunctions, disorders, and diseases [6,7,10,11]. Anti-aging medicine contributes to prolonging healthy life and reducing the chances of developing various age-related diseases, including intervening in the biological process of aging. In general, health-related policies focus on treatment after the onset of disease under the universal medical system, but there is a disadvantage of causing an increase in medical expenses. Therefore, the importance of medicine for various preventive approaches—namely, preventive medicine—is now emerging, and the paradigm of medical and health-related policies is changing. Anti-aging medicine, which focuses on aging, is also approached in various ways for preventive purposes, so besides playing a central role in the field of preventive medicine, it can also have a significant impact on improving the quality of life through life extension [6,12,13,14,15].

Moreover, while the primary approach in anti-aging diagnostic and medicine focuses on understanding and delaying the biological mechanism of aging, many of the mechanisms of the aging process remain unknown. Although it has been revealed through research in various fields that factors such as cell function degradation [4,16], oxidative stress of free radicals [5], decreased immunity [8], and changes in hormone levels [17] can affect aging, there are still some controversial aspects in terms of the results. As such, anti-aging medicine has been developing in various forms so far, pursuing biophysical and functional improvement of aging phenomena in our lives under the great goal of extending human lifespan and improving quality of life. This review explores different approaches and characteristics of anti-aging medicine and addresses the paradigms and future developments of technological developments in anti-aging medicine.

## 2. Methods

A comprehensive search was conducted to write this narrative review through PubMed and Google Scholar databases in December 2021. The keywords were “anti-aging”, “anti-aging medicine”, “anti-aging healthcare”, and “anti-aging diagnostic”. After removing duplicates from the search results in databases, the literature was selected for review. All searched papers were first screened by title and abstract. All human in vivo studies reporting diagnostic and healthcare approaches to anti-aging that were either original research or review papers containing the keywords mentioned above were included. Moreover, the worldwide aging population growth rate and future prediction were included to which studies referred regarding the reports of the United Nations. Since this study focused on the review of normal human anti-aging diagnostic medicine and prevention as the main content, research related to anti-aging in animals, anti-aging in humans with diseases, case reports, anti-aging approaches related to skin and beauty, and pharmacology (e.g., cosmetics, skin wrinkle improvement procedures, drugs manufacturing, etc.) were excluded.

## 3. Results

**Figure 1:**
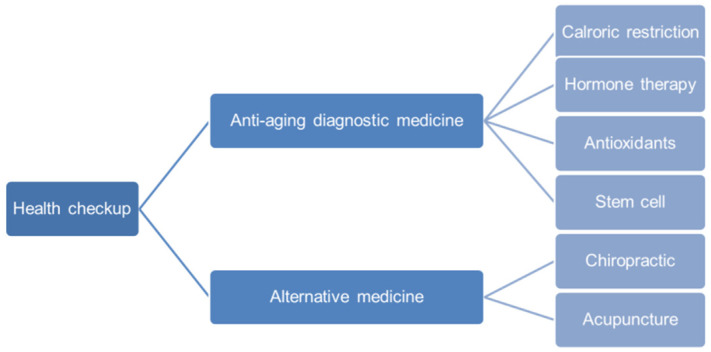
Various approaches for healthy life extension and anti-aging. Aging is difficult to judge in terms of appearance before all age-related diseases or pre-diseases become apparent. Therefore, it focuses on preventing the deterioration of health through periodic and systematic health checkups. In addition, since it is possible to induce an increase in function before complications and organ damage occur, through various approaches to anti-aging diagnostic medicine, it is the most effective method in the pre- or early stages of the disease.

Anti-aging diagnostic medicine mainly aims to help people enjoy their life comfortably through health and longevity. Even with the diseases, this does not necessarily lead to extreme death, impairing quality of life. Of course, people can enjoy health and longevity even if they have diseases. From this point of view, it is key to maintain health and a balanced state even if various physiological, biophysical, and pathological conditions occur to maintain a quality of life, which is an important aspect of anti-aging medicine. Up to now, many products, including a variety of diets, medications, and supplements, are marketed and promoted to have anti-aging benefits. Although there is currently no proven way to slow the aging process in humans even slightly, it is important to look at the various approaches for anti-aging and explore what may be needed in the future. In this review paper, various approaches, from basic health checkups to anti-aging diagnostic medicine and alternative medicine, are explored, as shown in Figure 1.

### 3.1. Anti-Aging Related Health Checkup

The health checkup related to anti-aging is a basic method to identify the signs and symptoms of aging by examining blood vessels, hormone levels [18], sensory function, and the balance of free radicals and antioxidant potential, in addition to the existing general comprehensive health checkup [19,20]. It is also a way to detect and deal with signs of physical and mental aging at an early stage [21]. These examinations make it possible to provide early detection and treatment to prevent aging-related diseases that can occur physically and mentally and guide lifestyle habits such as taking nutritional supplements, exercise programs, and stress control.

### 3.2. Caloric Restriction

Caloric restriction (CR), a well-known general dietary approach, is one of the ways to slow down human aging [22,23]. Although there are no conclusive studies that CR may cause anti-aging effects in humans, some results have suggested that it may benefit specific groups of people [24,25]. Fontana et al. found that CR had a protective effect on human atherosclerosis, and other studies reported beneficial effects on cardiac function [26]. CR can also improve memory in the elderly. In addition, Racette et al. found some benefits of CR for reducing body weight and obesity, although similar to those obtained with exercise, it suggests that CR has a beneficial effect on some biomarkers for longevity in overweight individuals [27,28]. However, side effects due to CR have also been reported. Mental stress caused by being hungry and the resulting depression and loss of libido are its representative effects [29].

### 3.3. Hormone Therapy

Many hormone levels decrease with age [30]. Growth hormone has a long history as an anti-aging agent, and some evidence suggests that growth hormone has beneficial effects in older people [31,32]. Growth hormone supplements can increase muscle mass, strengthen the immune system, and increase libido [33]. Previous studies have shown that using human growth hormone as an anti-aging treatment in older adults indicated that the risks of growth hormone far outweigh the benefits [34,35]. The drawbacks of using growth hormone are the occurrence of soft tissue edema and the potential possibility to stimulate the growth of cancer cells due to the effects of hormone growth stimulation [36]. Insulin-like growth factor 1 (IGF-1) [37], hydroepiandrosterone (DHEA) [38,39], and melatonin [40,41] are hormones whose production declines with age. Many studies have been performed on these types of hormones that can contribute to the prevention of aging. In addition, as regards the side effects, the occurrence of acne was reported, and one study in older women found no evidence of benefits from DHEA [38]. Although many studies have been performed on the preclinical side and have pointed out that it can help reduce aging-related diseases and extend lifespan, the lack of clear evidence for the roles of these hormones in humans anti-aging and occurrences of side effects in humans indicate that the use of hormones is not yet sufficient.

### 3.4. Antioxidants

Many cohort studies have reported that increased intake of dietary antioxidants [42,43], including vitamin E, vitamin C [44,45], and beta-carotene, is associated with a reduced risk of atherosclerosis-related diseases. Moreover, the effects of vitamin E on patients with Alzheimer’s disease showed significant differences in both antioxidant and cognitive function improvement abilities [43]. On the other hand, a randomized controlled trial of more than 35,000 healthy women over 45 years of age did not show any beneficial effects of vitamin E in preventing major cardiovascular events [45]. Although these antioxidants affect certain age-related diseases and medical advances for antiaging are being pursued, it is also necessary to recognize that the results are hard to generalize to the entire range of aging, and many researchers agree that continuous follow-up studies are necessary.

### 3.5. Stem Cell

In recent years, stem cells have received widespread attention since the possibility of treating aging diseases rejuvenation is interesting to researchers [46]. However, although the depletion and dysfunction of stem cells are thought to play a role in aging [47,48], it is still hard to mention that stem cell-based anti-aging treatments are effective because harvesting and/or preparing stem cells for treatment is complicated, and scientific research still remains to optimize the protocol. Nevertheless, in some parts, stem cells have been shown to be useful, such as blood and bone marrow-derived stem cells used in some autoimmune and cardiovascular diseases [49]. However, stem cell applications are still in the early stage and still far from allowing physicians and doctors to use stem cells to delay aging.

### 3.6. Alternative Medicine

Alternative medicine is a type of antiaging medicine that is easily accessible to the elderly [50]. Since alternative medicine is rarely taught as part of traditional medical school education systems, it often does not meet the mainstream medical community and standards [51,52]. Nevertheless, in 1993, Eisenberg et al. pointed out that 43% of Americans use certain forms of alternative medicine, such as chiropractic, massage, and relaxation therapy [49]. In particular, many previous studies have reported that alternative medicine in the elderly is much higher [53,54,55]. Notably, the therapeutic form of alternative medicine appears differently depending on the ethnic background. Asians are more likely to receive acupuncture, whereas Hispanics are more likely to receive dietary supplements, and Caucasians are more likely to receive chiropractic care [56,57]. Alternative medicine, sometimes referred to as Chinese medicine or oriental medicine, is commonly referred to as complementary and alternative medicine, which refers to a more collaborative than an adversarial relationship with conventional medical practice.

Chiropractic is the most commonly used alternative medicine modality and uses treatments that focus on chiropractic and manual techniques for patients with musculoskeletal symptoms [58]. Massage stimulates the skin, muscles, and nervous system to induce relaxation and reduce stress. Each type of massage gives a unique pressure, friction, repetitive force, and stroke pattern. Although chiropractic and massage have not demonstrated significant advantages over physical therapy and other medical treatment methods, they are mainly used by the elderly due to their ease of access and simple concept of exercise treatment.

Acupuncture and acupressure have been used as unique treatments in the Orient. The manipulation of multiple meridian energy pathways in the body through needles and acupressure aims at balancing the opposite negative and positive forces, thereby stabilizing the energy. Acupuncture is best known for its effects on chronic pain and substance abuse management [59]. However, it is difficult to secure the homogeneity of several acupuncture-related studies. The outcome effect of acupuncture is still controversial to provide clear evidence in its application, such as mixed results in each analysis, except that one study clearly showed clinical improvement [60]. Although it is currently accepted by Western medical practitioners and practiced by some pain management physicians in the United States, it still remains challenging to provide a clear scientific or clinical basis for treatment.

Complementary and alternative medicine has been developed alongside clinical medicine due to the craving desire to delay the aging process and treat diseases in humans. Many people use complementary and alternative medicine because it works better than clinical medicine in some cases. In addition, complementary and alternative medicine also has the advantage of providing patients with a level of time, contact, attention, and personal interaction that is not increasingly common in modern medicine. However, large and well-designed studies are needed to test the effectiveness of alternative medicine. As in other medical fields, there is a constant shortage of research on the elderly. As interest in complementary and alternative medicine grows, healthcare providers need to remain open to both the potential benefits and potential risks of alternative treatments. If treatments prove to be dangerous or ineffective, we need to educate the general public and strive to remove these treatments from the market. Once the treatment is proven effective, Western and Eastern care providers need to work with patients to provide the most appropriate and comprehensive medical services.

## 4. Discussion

**Figure 2:**
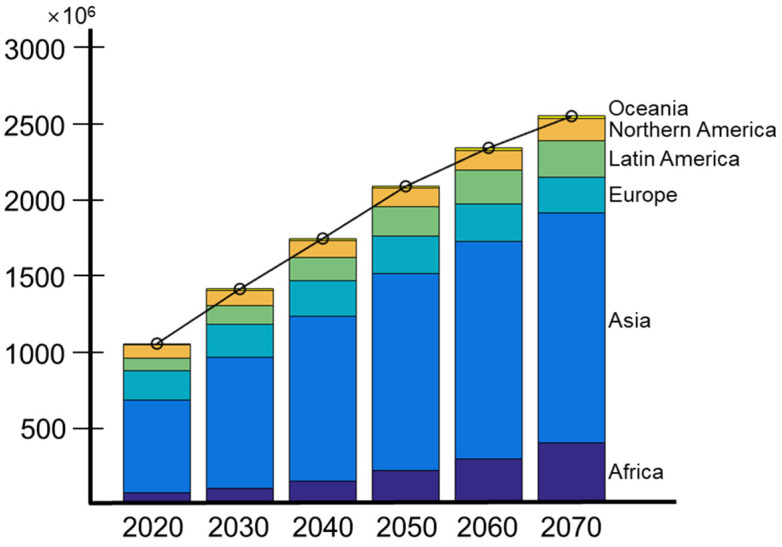
Probabilistic projection of population age 60+ (both sexes combined) by geographic regions from 2020 to 2070 with median (50 percent) prediction interval.

The aging population and low fertility issues may increase the medical cost burden of the National Health Insurance, and policy adjustments may cause another financial/economic problem that individuals may face. Population aging is occurring much faster than in the past, and the proportion of the world’s population over the age of 60 is projected to nearly double, from 12% to 22%, between 2015 and 2050 [61]. In addition, the United Nations (UN) report, which is related to the probabilistic prediction of the increase in the population aged 60 and over by continent, indicates that the aging population can continue to increase by about 2.5 times from 2020 to 2070 (Figure 2) [62].

Therefore, countries worldwide are faced with the challenge of building and utilizing health and social systems to deal with this situation. However, if many older people are placed in an environment where they can stay healthy and work, the burden of medical costs could be maintained at current levels. Therefore, interest in antiaging medicine has become an issue that can only be further increased, and it is an important accompanying problem that solutions can be presented in various ways. In addition, as the aging of the population raises public interest in various consultations and support for aging management, antiaging medicine can contribute to helping to solve the environmental, policy, and public issues caused by the current aging population. As humans age, the framework for classifying aging process awareness can be considered natural phenomena and disease processes. Scientists who regard it as a disease process are attempting to improve humankind’s overall health and longevity through various studies, beginning with the molecular origins of age-related diseases. Persons in favor of natural phenomena recommend trying various physical and mental improvement efforts as aging progresses.

Aging is a natural phenomenon that humans have no choice but to accept its irreversibility over time. As a result, humans are more focused on living a healthy life and improving their quality of life, despite persistent aging. The primary purpose of antiaging is to extend healthy life. A variety of biological factors—the environment, lifestyle, and health care system—are fundamental factors in improving health and moving away from the causes of disease and death. In general, changes in an individual’s lifestyle that can be easily applied, such as active physical activity and exercise, improvement of nutrition, and the help of an advanced medical system and medical services, are important features of antiaging. Although the various methods covered in this review are presented as solutions to solve the antiaging issues with the aforementioned fundamental factors, they still have some disadvantages that could not be ignored for each method, so it is difficult to generalize or assert strong effects for helping elderly individuals actively. Nevertheless, many approaches in antiaging medicine constantly strive to overcome shortcomings and achieve better antiaging medicine. These factors are important research themes for researchers and direct links to the development of antiaging medicine.

The American Academy of Antiaging Medicine (A4M) believes that “changes in antiaging” can be produced by a combination of interventions that include hormones, antioxidants, lifestyle modifications, and exercise [63]. These include slim body maintenance, smoking cessation, regular exercise, active social and sexual life maintenance, sustained psychostimulation, stress avoidance, healthy eating, and moderateness, emphasizing several widely recommended interventions to maintain the health of the elderly, such as a healthy diet. A4M argues that these interventions lead to antiaging and can also extend the human lifespan. In addition, with continued scientific developments such as nanotechnology and stem cell therapy, it is predicted that humanity will evolve into an ageless society in which we all experience endless physical and mental vitality.

The most basic method for antiaging is appropriate physical activity or exercise. It is a method that can be easily applied regardless of a particular tool or place and can induce an increase in the functions of the endocrine system and the immune system by preventing or delaying the decrease in physiological function, muscle function, digestive function, and metabolic rate. Furthermore, in addition to biophysiological effects, it is possible to achieve positive effects emotionally and psychologically, to help the elderly avoid depression and anxiety and gain confidence through physical activity and exercise. Second, it is possible to obtain preventive effects on various physical and mental illnesses through stress management. Stress can lead to inappropriate forms of health, such as chronic drinking and increased smoking due to negative emotions, leading to various illnesses, including cardiovascular disease. Ultimately, failure to manage stress is detrimental to an individual’s aging progression.

Another factor facing the challenges of developing antiaging medicine is the ethical issues that may arise from economic and cultural approaches [64,65]. It is possible to have critical perspectives on the commercialization of technology for life extension and the social problems of overpopulation caused by antiaging medicine. Moreover, from the opposite point of view, some researchers highlight the positive perspectives of anti-aging—namely, antiaging medicine can potentially reduce the incidence of many diseases and enable a healthy life. Therefore, it is necessary to reflect critically on the socioeconomic issues and the particular problem of the antiaging medicine market and consider the issues of inequality that may arise from these issues. Ultimately, this is an important aspect that requires continuous reflection on the legitimacy and process of medicine based on a scientific approach to solving the problem of aging.

## 5. Conclusions

Multiple studies demonstrated that antiaging medicine and healthcare are essential factors in leading a healthy and active life for the elderly population. Nevertheless, since many conditions may be undiagnosed and undertreated, among the various forms of antiaging medicine and healthcare, many older people should decide to care for their own needs. While older people can easily start managing antiaging in terms of a simple health checkup, a more detailed view on anti-aging medicine and healthcare extends beyond simple clinical medicine, and such practices are being provided in various forms to meet the needs of the elderly with alternative medicine and biophysical technology. All findings elaborated in this review are expected to promote healthy aging further. In addition, toward the fundamental development of antiaging medicine, future plans need to be presented to actively utilize multiple antiaging approaches and economic, medical, and ethical considerations arising from the relationship between the increase in the elderly population and the continuous development of antiaging medicine should be considered. It is believed that primary diagnosis and preventive approaches to antiaging can further be developed only when harmonious collaboration is achieved.

## Data Availability

Not applicable.
